# Surgery for Cutaneous Squamous Cell Carcinoma and its Limits in Advanced Disease

**DOI:** 10.5826/dpc.11S2a167S

**Published:** 2021-10-01

**Authors:** David Moreno-Ramírez, Francisca Silva-Clavería, Almudena Fernández-Orland, Noemí Eiris, Andrés Ruiz de Casas, Lara Férrandiz

**Affiliations:** 1 Department of Medical-&-Surgical Dermatology. University Hospital Virgen Macarena. Medicine School, University of Sevilla. Seville, Spain.

**Keywords:** cutaneous squamous cell carcinoma, surgery, oncologic surgery, Mohs surgery, nodal surgery, nodal metastases

## Abstract

Surgery remains the first-line therapeutic option for most patients with cutaneous squamous cell carcinoma (cSCC). However, in the current therapeutic landscape, surgery must attempt to the complete tumor resection (R0 resection) with the lowest risk of surgical complications. This double aim is usually accomplished through standard excision with clinical margins in patients with low-risk tumors or by some of the micrographically controlled surgery procedures for patients with tumors at high-risk of local recurrence and metastasis. Surgery is also a first-line treatment for nodal metastases of cSCC as well as an option to consider in patients who develop recurrences while receiving immunotherapy, or as a palliation procedure in patients with advanced tumors. Neoadjuvant immunotherapy, that is the use of a medical treatment before surgery, is under investigation in patients with cSCC. The decision-making process and guidelines recommendations regarding cSCC surgery are reviewed in this manuscript.

## Surgery: Still the Front-Line Treatment for Cutaneous Squamous Cell Carcinoma

The therapeutic landscape for non-melanoma skin cancer has evolved significantly in recent years. However, surgery remains the first-line therapeutic option for most patients with non-melanoma skin cancer, which includes basal cell carcinoma and cutaneous squamous cell carcinoma (cSCC) [1,2]. Although no clinical trial or systematic reviews have addressed the effectiveness of surgery for cSCC, most primary tumors can be safely approached through surgical excision [2]. Surgical excision offers high-cure rates and long-term control of primary cSCC and is indeed the treatment of choice for most patients with cSCC, as also recommended by international guidelines [2–4,6].

Given the leading role of surgery for cSCC and the need for individualized approaches for patients, this article addresses the surgical issues that are under debate in the literature and in multidisciplinary tumor boards.

## What is Appropriate Surgical Excision?

### Aims of cSCC Surgery and Appropriate Surgery for Primary Tumors

R0 surgery, that is the clinical and complete microscopic resection of the tumor, is the main goal of oncologic surgery. With the current availability of non-surgical therapeutic options for cancer, however, this goal should be adapted based on the oncologic effectiveness and the patient’s acceptance in terms of morbidity. Thus, appropriate surgery for cSCC has the objective to achieve R0 resection but also to preserve function and quality of life as much as possible [5]. If all these criteria cannot be met via surgical procedure, alternative therapies, such as radiation therapy and systemic therapy, should be considered.

**Figure 1:**
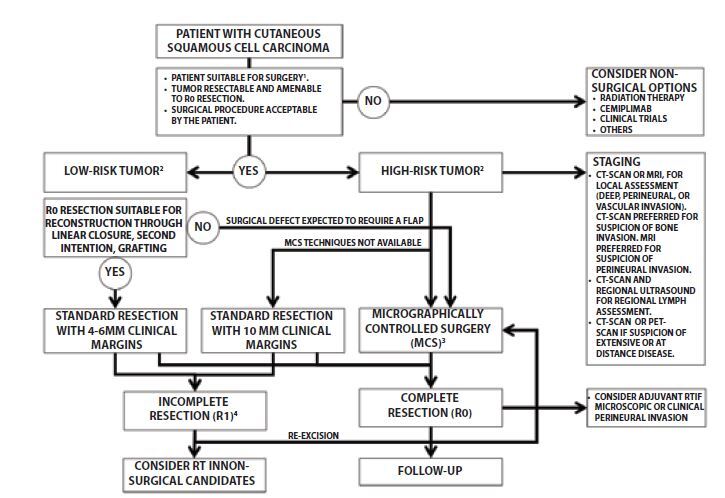
Decision algorithm for the surgical management of patients with primary cutaneous squamous cell carcinoma. (A) ECOG-PS 0-2, acceptable overall condition, lack of non-controlled major cardiovascular or hematologic morbidities. (B) Assessment of accepted high-risk criterion (Table 1). (C) Micrographically controlled surgery always preferred as first option in high-risk tumors. Intraoperative frozen-section assessment or paraffin-embedded sections with delayed closure techniques based on tumor-, patient-related features, and availability of the procedures. (D) For definition of incomplete resection see the text.

Decisions on the most appropriate surgery for cSCC should start with a comprehensive assessment of the tumor’s clinical and pathological features. These tumor features, in addition to other patient-related characteristics, provide information on the risk of local recurrence and metastasis, helping to classify tumors into low-risk and high-risk groups (Table 1) (Figure 1) [2,4,6].

The optimal surgical procedure for patients with cSCC will fit the tumor’s risk profile, and the therapeutic goals (R0 surgery) can be accomplished through standard surgical resection with clinical margins, or through micrographically controlled surgery (MCS) (Figure 1) [2–4].

### Standard Excision with Clinical Margins

Standard excision with clinical margins and a postoperative pathological evaluation of the margins are recommended for primary cSCC without high-risk features and for patients with high-risk tumors who are not suitable for or cannot access MCS [3, 4, 7]. In general, retrospective analyses, prospective observational studies and pooled analysis of observational studies on standard surgery with clinical margins of head and neck cSCC, have reported 5-year recurrence-free survival rates above 90% and recurrence rates below 6% [8, 9].

The appropriate clinical margins to be applied have been explored in various studies assessing the cure rates of a variety of margin thresholds based on tumor risk features. Accordingly, 95% of tumors < 2 cm with well-defined borders have been reported to be successfully managed with 4 mm clinical margins, whereas tumors > 2 cm require clinical margins of at least 6 mm to achieve histologically clear margins in 95% of cases [4]. If additional high-risk features are present, the clinical margins can increase up to 9 mm. In general, the larger the tumor the higher the number of tumor risk factors (eg, poor differentiation, high-risk location, perineural invasion), and the wider the clinical margin to be applied.

The European Association of Dermato-Oncology (EADO) guidelines recommend clinical margins of 5 mm for low-risk cSCC and 6–10 mm for those tumors with accepted high-risk features, provided that MCS is unsuitable or unavailable [3]. The National Comprehensive Center Networks guidelines recommend 4–6-mm clinical margins and postoperative margin assessment for low-risk tumors, whereas wider margins are preferred for high-risk tumors. However, these guidelines do not specify the clinical margins to be applied for high-risk tumors due to the variability that these tumors encompass and therefore recommend individualized margins adapted to tumor and patient-related factors [11]. Table 2 shows a summary of these recommendations.

In contrast to lateral clinical margins, the current guidelines offer no concrete recommendations for managing deep margins beyond including the subcutaneous tissue while sparing the perichondrium or periosteum, provided that there is no clinical involvement of the aforementioned structures. [3]. This recommendation applies to ear cSCC. If the perichondrium is not clinically involved, the structure should be kept untouched, representing the deepest margin of resection. Subcutaneous tissue should be resected, particularly when dealing with ear and scalp tumors, up to the periosteum or perichondrium level, sparing these structures if not clinically involved. However, a recent study analyzing the rate of incomplete excision in cSCC showed that residual disease was located at the depth of the surgical specimen rather than in the lateral resection margins [7]. In this study, the overall incomplete excision rate was 7.6%, with 94% of incomplete excisions involving the deep margin. These results led the authors to suggest that if intraoperative frozen sections are not performed, superior deep clearance can be achieved by excising an extra deep fascial plane of tissue, even in the presence of a macroscopically clear deep plane [7].

### Micrographically Controlled Surgery

Micrographically controlled surgery (MCS) involves the intraoperative examination of the tumor’s resection borders through frozen sections. This is done to confirm, the tumor’s complete removal, prior to the incision closure [12]. MCS also avoids the unnecessary removal of uninvolved tissue, which is important for tumors located in critical anatomical sites [3,12]

Mohs micrographic surgery (MMS) was the first technique developed to meet the aims of ensuring complete tumor removal and avoiding the unnecessary excision of healthy tissue [13]. Since its introduction, MMS has been considered the first-line surgical procedure for locally invasive, high-risk skin cancers’ removal. MMS is especially useful when maximal preservation of unaffected tissue is essential. Classic MMS is a day surgery procedure performed under local anesthesia and involves the following steps: mapping the procedure, debulking the primary tumor, tissue layers’ excision, frozen section processing and analysis, re-excisions of further tissue from involved areas, and reconstruction of the surgical defect. The most common cancers treated with MMS are basal cell carcinoma and cSCC, although MMS is also employed to remove other skin cancers such as dermatofibrosarcoma protuberans, Merkel cell carcinoma, and lentigo maligna.

With respect to cSCC, a prospective multicenter case series showed a 5-year recurrence rate after MMS of 3.9%. The recurrence rate was 2.6% in patients with primary SCC and 5.9% for patients with previously recurrent SCC (P < 0.001). In view of this low 5-year recurrence rate, the authors emphasized the importance of margin-controlled excision for SCC. A recent retrospective cohort study of patients with a SCC treated with MMS or standard excision also showed an 8% recurrence risk after standard excision, higher than the 3% after MMS, and a higher cumulative incidence of recurrence for standard excision than for MMS during the entire follow-up period. Carcinomas treated with MMS were at a 3-fold lower risk of recurrence than those treated with standard excision when adjusted for tumor size and deep tumor invasion (adjusted hazard ratio (HR) 0.31, 95% confidence interval 0.12–0.66) [13, 14, 15].

In cSCC, however, tumor extensions can be better assessed in paraffin sections, and there is the likelihood of false-negative results in frozen sections. Paraffin-embedded section assessment with deferred closure has therefore been favored for patients with high-risk cSCC, using techniques that allow for complete circumferential peripheral and deep circumferential margin assessment (CCPDMA or 3D surgery) [4, 12, 16, 17]. These procedures are also particularly appropriate for patients with tumors requiring general anesthesia. Table 2 provides a summary of the recommendations for MMS and other MCS, CCPDMA and 3D surgery for cSCC.

### Surgical Defect Reconstruction

Another essential issue after conventional cSCC surgery is the reconstruction procedure. Appropriate closure after cSCC resection provides proper tissue coverage of the surgical defect, restores the function and cosmetic appearance of the anatomical region, and allows for early detection of potential local recurrence. These are particularly relevant issues for tumors removed through conventional surgery with clinical margins, a procedure that, as mentioned, cannot ensure the complete removal of the tumor. Consequently, any reconstruction technique that involves tissue movement or rearrangement, particularly rotation, or that provides thick coverage of the surgical defects should be avoided if clear resection margins are not histologically confirmed [3]. After standard surgery with clinical margin resection and linear closure, second intention healing and thin skin grafting are the preferred closure procedures [3,4]. If local flaps or more complex reconstruction techniques are expected, an intraoperative surgical margin assessment is essential (Table 2).

## When Primary cSCC Surgery Fails

### Management of R1 Tumor resection

Standard resection with clinical margins and postoperative microscopic control of the margins entails the major risk of incomplete resection. Depending on the study, incomplete excision (R1 resection) has been defined for the standard vertical bread-loaf technique as follows: the presence of tumor cells at the surgical lateral or deep margin, the presence of residual tumor within 0.5–1 mm or “close to” the margins of the excised specimen, or a tumor-free margin ≤ 2 mm [14, 19, 20].

A recent systematic review showed an overall incomplete excision rate of 13% for cSCC on head and neck, and other body locations [20]. Head-and-neck locations, tumor depth and size, invasive growth, and re-excision were indicated as the risk factors for incomplete excision [20]. Another study identified perineural invasion due to subclinical spread below and beyond the cutaneous margin as a predictor of incomplete resection [19].

The need for re-excision of incompletely excised non-melanoma skin cancers has been a matter of debate. Incomplete excision of cSCC leads to an increased risk of local recurrence, deep subclinical progression, and metastasis, prompting current guidelines to recommend re-excision of those cSCCs with positive resection margins, particularly with deep margin involvement, except for patients unwilling or unfit to undergo another surgical procedure [3,4]. Re-excision of incompletely resected tumors often yields clean margins. Re-excision specimen might however not contain tumor cells and still, there is evidence for up to 5% of patients with negative re-excisions who developed local recurrence [18–20].

Due to the methodological issues related to the heterogeneous concept of incomplete excisions, the rate of residual tumor cells in re-excision specimens ranges from 29% to 100% [19, 20]. Additionally, there is evidence of a lower degree of differentiation in re-excision histology reports compared to the primary excision specimen [19].

As with primary surgery, cSCC should also be adapted to the tumor’s risk profile. Regardless of the tumor’s risk, however, an MCS procedure with frozen or permanent sections is preferred as the surgical option for incompletely resected cSCC. If not available or if the patient is unsuitable, patients with low-risk tumors can be managed through standard re-excision with postoperative margin assessment. Appropriate clinical margins for these re-excisions have not been defined but should be based on the extension of the primary specimen’s margin involvement, after considering tissue shrinkage during the process [3,4]. Patients with incompletely resected high-risk tumors should always undergo an intraoperative or delayed MCS procedure (Table 2).

## Surgery Beyond Primary cSCC

### Surgical Management of Lymph Node Metastasis of cSCC

In patients with cSCC, regional nodal disease represents a major event in up to 4% and 6% of patients overall, a rate that increases if the primary tumor is at high risk and is in an advanced stage [21, 22]. However, the survival of patients with nodal metastases is not necessarily poor. Five-year disease-specific survival for patients with low-burden single nodal metastasis (stage I) is approximately 90%, a survival rate that falls to 75% and 42% for patients with multiple and large-burden metastases (stage II and III, respectively) [21]. Given that these results have been classically achieved through lymph node dissections, it appears that regional lymph node surgery still plays a role in the routine management of nodal regional disease.

However, most of the available literature related to nodal surgery of cSCC refers to head-and-neck tumors, with few references to tumors in other body sites. There is a lack of high-quality studies, randomized clinical trials, and large prospective cohort studies. Recommendations on managing regional nodal basins are therefore mostly based on low-to-medium levels of evidence [3].

### Sentinel Lymph Node Biopsy in Patients with cSCC: Is it Worth Performing?

To date, there have been no randomized clinical trials that have assessed the role of sentinel lymph node biopsy (SLNB) in patients with cSCC in terms of survival, regional control, or any other outcome. The available evidence comes from a number of small prospective series and systematic reviews of retrospective studies that have reported a positive SLNB rate of 12%–17% [23–26], rates below the rates of positive sentinel lymph nodes in patients with melanoma and intermediate thickness tumors (16%–20%), a subgroup of patients with melanoma for whom SLNB is the standard of care and a widely recommended procedure [27, 28]. In terms of survival outcomes, the reported results on the prognostic ability of SLNB for patients with cSCC are also conflicting, with a number of studies showing improved disease-specific survival in SLNB-negative patients, whereas other series have failed to demonstrate any survival benefit in patients without microscopic nodal disease when compared with those with positive SLNB [24, 29, 30]. SLNB eligibility for patients with cSCC is usually determined by 2 additional clinical features that frequently coincide in these patients: age and anatomical location. Between 75% and 90% of these tumors originate at the level of the head and neck, an anatomical location where surgeons faced specific challenges when compared with, for instance, trunk and limbs. Head and neck tumors usually have more complicated lymphatic drainage patterns, with a high frequency of bilateral and contralateral drainage. In this region, lymph nodes are more often tiny and usually overlap each other. This makes anatomical and gammagraphic inspection/identification more difficult compared with other anatomical locations [31]. Head and neck melanomas, for instance, are well known to be associated with the non-visualization of sentinel lymph nodes (SLNs) on lymphoscintigraphy, higher false negative SLNB rates, and lower SLNB positivity [32]. Moreover, the surgical anatomy of the neck is challenging, and, in the case of the parotid gland where 70% of head and neck SCCs drain, its relationship with facial and accessory nerves requires a thorough analysis of the risk-benefit balance. The literature on SLNB in patients with melanoma has also shown that increasing age is related to lower SLNB positivity rates, slower lymphatic drainage, and greater surgical risk related to the poorer performance status of elderly patients [31, 33].

For all these reasons, the currently available results, the poor evidence, and other technical and clinical issues, the current guidelines do not recommend SLNB as a routine procedure for managing cSCC patients, except for clinical trial settings (Table 3).

### Complete Lymph Node Dissection: An Opportunity to Preserve

When left untreated, nodal disease is necessarily progressive and can become distressing and life-threatening for cSCC patients. Accordingly, the guidelines definitely recommend performing therapeutic lymph node dissection as a routine procedure in patients with nodal recurrence, detected either clinically or by imaging [3,4]. Evidence supporting this recommendation is not outstandingly strong, as this is based on a single prospective series, several retrospective studies, and systematic reviews of these studies, all of which analyzed exclusively cutaneous head and neck tumors [35, 36].

**Figure 2:**
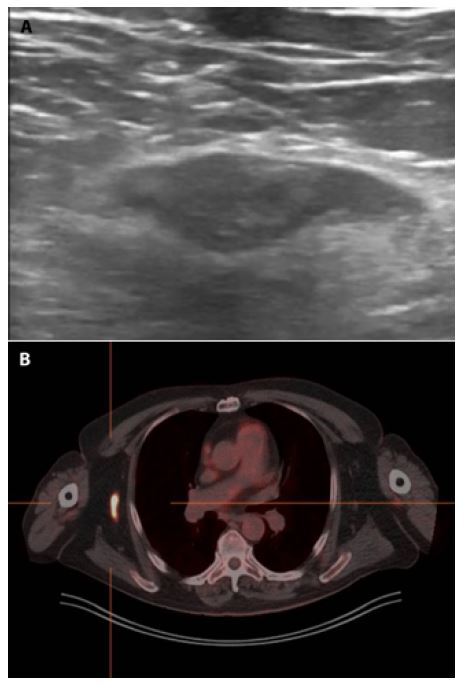
Lymph node metastasis of cutaneous squamous cell carcinoma on the right arm. (A) Regional ultrasound shows a 17 mm hypoecoic structure also identified in the PET-CT scan. (B) The patient undewent right axillary lymph node dissection.

**Figure 3:**
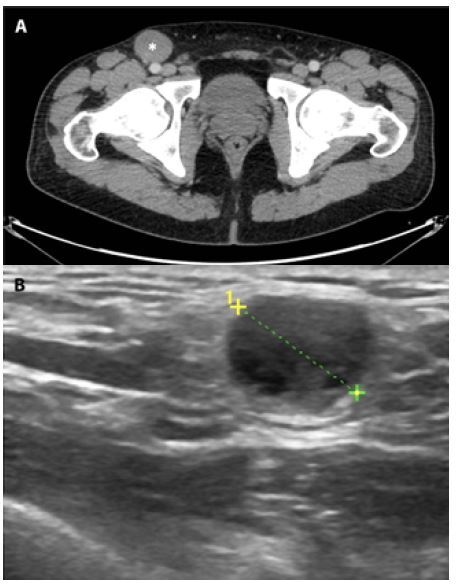
Lymph node metastasis of cutaneous squamous cell carcinoma from a primary tumor on the right sole. (A) CT-scan shows a well defined 20 mm nodule on the right superficial groin (white asterisk). (B) Regional ultrasound showed an anecoic rounded structure. The patient underwent a groin lymph node dissection.

Nevertheless, skin cancer clinics should focus their efforts on the early detection of lymph node metastases for possible surgery of low-burden metastatic disease, with the expectedly lower surgical morbidity. Close follow-up of regional basins using ultrasound has gained interest as a routine imaging procedure for the early detection of nodal metastasis (Figure 2–3) [4].

Another issue for patients with nodal metastasis is the appropriate extent of the dissection. Patients with cSCC and nodal involvement have usually undergone complete radical lymph node dissections of the involved regional basin, which involves the 3 levels of the axillar basin, the superficial and deep groin nodes, and the 5 levels of the neck. Additionally, complete dissections in the neck are often completed with superficial parotidectomy if the parotid gland is affected.

However, over the last decade, a trend towards the consideration and offer of less extensive and more selective lymph node dissections has developed, with cSCC patients. The few available studies on selective neck dissections have shown regional control and survival rates of 85%–100%, rates similar to those reported for conventional radical and modified radical neck dissections [37, 38]. Thus, selective neck dissection appears to provide an oncologically effective and safe surgical procedure for those patients with clinically positive nodes in the neck and with no other high-risk clinical feature. This also applies to patients with low or intermediate nodal burden, non-fixed or with no muscles or major vessel invasion, although these features should unfailingly lead to radical and complete neck dissection. To date, there are still no studies on selective groin or axillary dissections in patients with cSCC.

In any case, the extent of lymph node dissections should be discussed and determined by the surgical team in the context of an interdisciplinary tumor board and after a thorough assessment of tumor-related (aggressiveness, involved regional basin, tumor burden, etc.), surgical (potential complications, morbidity, etc.), and patient-related features (overall condition, performance status, preferences, expectations, etc.). Table 3 shows a summary of the recommendations for the surgical management of lymph node regions.

## When Not to Operate. The Limits of Surgery for Patients with cSCC in 2021

### Surgery for Patients with Advanced cSCC

In line with the famous quote “the best surgeons are those who know when not to operate”, major and radical procedures causing major anatomic mutilation or physical disfiguration, in an attempt to achieve oncological results, should no longer be first-line options for cSCC patients [39]. Thus, if there is a clinical situation in which the role of surgery needs to be revisited, such as for other skin cancer types, it is that of patients with advanced disease.

**Figure 4:**
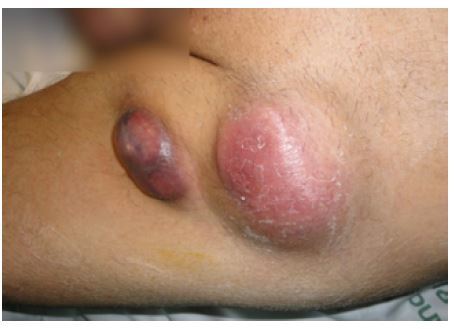
Advanced cutaneous squamous cell carcinoma (cSCC). A 70-year old man with unresectable lymph node metastasis on the groin from a previously resected high-risk cSCC arising on a previously radiated area on the left heel.

**Figure 5:**
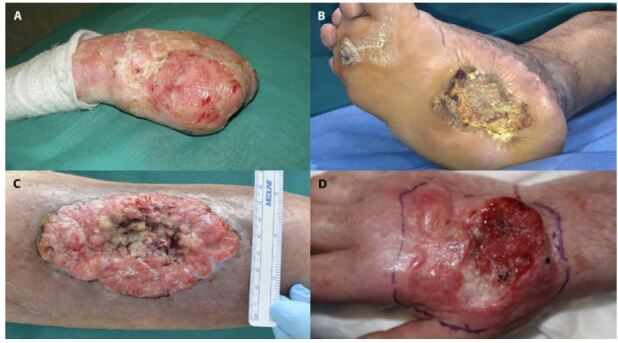
Advanced cutaneous squamous cell carcinoma (cSCC). (A) A 32-year old man with dystrophic epidermolysis bullosa who developed an unresectable cSCC over a chronic wound on the left hand stump. The patient underwent amputation. (B) A 50-year old man with polyomelitys who developed an unresectable cSCC over a chronic ulcer on the right sole. The patient refused radiation therapy and systemic immunotherapy and a lower leg transtibilial amputation was carried out. (C) A 70 year-old woman who developed a neglected 10-year history ulcer on the right leg tha was considered unresectable. Radiation therapy achieved partial response and knee disarticulation had to be performed. (D) A 70-year old man immunosuppresed due to kidney grafting who developed fast-growing ulcer on the right hand. Systemic immunotherapy and radiation therapy did not achieved clinical response and consequently the patient underwent major amputation.

Advanced cSCC usually encompasses the following 2 clinical contexts: 1) unresectable primary, recurrent, or metastatic tumors due to a large tumor burden, invasion of major vessels, neural or bone underlying structures hampering R0 resection, and 2) tumors or metastasis for which complete resection unfailingly entails a major anatomical defect, or a functional or cosmetic impairment that is unbearable for the patient (Figure 4). These clinical contexts usually present in patients with additional conditions, favoring the tumor growth over long periods before seeking the needed care (neglected patients, lack of caregivers, etc.), or render patients more prone to aggressive invasion due to immunosuppression (organ recipients, lymphoproliferative conditions, etc.), genetic disorders (ie xeroderma pigmentosum), or local factors (ie previous radiation therapy and burns) (Figure 5).

Considering the current therapeutic landscape, the presence of these criteria for advanced cSCC should be accepted as the real limit for surgery as front-line therapy for patients with cSCC. Accordingly, the current recommendations on these clinical settings indicate radiation therapy, systemic therapy with the recently approved anti-PD1 antibody cemiplimab, and clinical trials as first-line therapeutic options for patients with advanced cSCC [3,4].

However, there are still 3 situations for patients with advanced cSCC in which surgery is likely to play a significant role. The first is when the patients are undergoing immunotherapy or other systemic therapies and develop further resectable recurrences. Determining the appropriate therapy for those cancers for which immunotherapy is available should be a dynamic process far from the classical binary approach based on deciding between surgery versus chemotherapy. The experience gained with other tumor types treated effectively with immunotherapy (eg malignant melanoma) provides insight into the capability of surgery to completely remove recurrences (mainly regional), while keeping distal disease under control through systemic immunotherapy. Although a survival benefit is not expected from surgery for these patients with recurrent disease, it might help the patients restore impaired function and quality of life.

The second situation is when surgery may be considered in the palliative setting, although as a last resort. Major resections, including major limb amputations, might be acceptable and are still performed from time to time on patients with untreatable and unbearable pain and unmanageable bleeding. The only aim of surgery in this clinical situation is to reduce the symptoms. Surgery should therefore not be offered if these symptoms can be controlled through other non-surgical options. However, in the case on minor amputations (ie finger or toe amputations) this radical surgical approach may leave the patient free of disease, providing long recurrence-free and overall survival.

Finally, the use of systemic immunotherapy in an attempt to reduce tumoral burden, thereby allowing for a less extensive surgery (neoadjuvant therapy), is being assessed in ongoing clinical trials on advanced cSCC. A recently published phase II pilot trial of neoadjuvant immunotherapy with cemiplimab has shown pathologically complete responses in 70% of the patients [40]. Although these results were obtained from patients with advanced but resectable tumors, it can be hypothesized that the same neoadjuvant approach can be applied to borderline resectable or even unresectable advanced cSCC in the future.

## Improving the Results of Surgery

### Adjuvant Radiation Therapy for Primary and Metastatic cSCC

The risk of residual disease after inadequate or incomplete surgery of high-risk primary tumors and lymph node metastases is usually managed through adjuvant radiation therapy. The benefits and indications of adjuvant radiation therapy for patients with cSCC is beyond the scope of this article. For patients with primary or nodal metastasis with high-risk features, however, surgery may be maintained or enhanced by postoperative radiation therapy. Therefore, for those patients with incompletely resected primary high-risk cSCC and those with completely excised but aggressive nodal metastasis (eg large burden, extracapsular extension), or incompletely excised involved nodes that are not suitable for further surgery, radiation therapy should be discussed and offered [3,4]. This essential part of cSCC management will be comprehensively addressed elsewhere in this monograph.

## Conclusions

Appropriate surgery for patients with cSCC represents a challenge in terms of oncological outcomes, postoperative function, and quality of life. Successfully accomplishing this task is not just a matter of surgeon expertise or technical procedural aspects. Oncologically successful surgery for patients with cSCC requires timeliness, proper surgical procedures based on guidelines and tailored to the patient’s clinical condition and the tumor’s particular features and, above all, requires to be acceptable to the patient.

Skin cancer clinics and multidisciplinary tumor boards should strive to meet the requirements for cSCC proper surgery procedures. If the criteria are met, surgery coupled with the recent breakthroughs in systemic immunotherapy is likely to offer patients with cSCC the best standard of management, proving longer survival and greater quality of life.

## Figures and Tables

**Table 1:** Clinical and Pathological Criteria for the Definition of Cutaneous Squamous Cell Carcinoma at High-Risk of Recurrence

**EADO Guidelines [6]**	Tumor diameter > 20 mm Localization on temple, ear, lip area Thickness > 6 mm or invasion beyond subcutaneous fat Poor grade of differentiation Desmoplasia Microscopic, symptomatic, or radiological perineural invasion Bone erosion Immunosuppression
**NCCN Guidelines [4]**	Size 2 cm to < 4 cm on the trunk, extremities Head, neck, hands, feet, pretibial, and anogenital (any size) Poorly defined Recurrent tumor Immunosuppression Site of prior radiation therapy or chronic inflammatory process Rapidly growing tumor Neurologic symptoms Histologic features: Acantholytic (adenoid), adenosquamous (showing mucin production), or metaplastic (carcinosarcomatous) subtype Perineural involvement
	Very high-risk: ≥ 4 cm (any location) Poor differentiation > 6 mm or invasion beyond subcutaneous fat Desmoplastic SCC Tumor cells within the nerve sheath of a nerve lying deeper than the dermis or measuring ≥ 0.1 mm Lymphatic or vascular involvement

**Table 2:** Guidelines Recommendations Concerning Surgery of Primary Cutaneous Squamous Cell Carcinoma (cSCC) Patients

**EADO Guidelines [3, 6]**	Low-risk cSCC should be excised with a clinical safety margin of 5 mm. High-risk cSCC should be excised with a clinical safety margin of 6–10 mm or by MMS/MCS. This margin should fall within the 6- to 10-mm range and be based on individual risk assessment and a constellation of tumor- and patient-related characteristics. As long as an R0 resection is not histologically confirmed, wound closure with local tissue movements (flaps) should be avoided. In case of positive margins, a re-excision shall be done, for operable cases.
**NCCN Guidelines [4]**	**Low-risk cSCC:** Standard excision with 4- to 6-mm clinical margins and postoperative margin assessment and second intention healing, linear repair, or skin graft. Closures like adjacent tissue transfers, in which significant tissue rearrangement occurs, are best performed after clear margins are verified Positive margins: Mohs micrographic surgery (MMS) or other forms of CCPDMA. Standard re-excision if clinically feasible. Negative margins: follow-up. **High-risk or very-high-risk cSCC:** MMS or other forms of CCPDMA (preferred for very high risk). Standard excision with wider surgical margins and postoperative margin assessment and linear or delayed repair. Due to the wide variability of clinical characteristics that may define a high-risk tumor, it is not feasible to recommend a defined margin for standard excision of high-risk CSCC. Positive margins: Re-resect, MMS or other forms of CCPDMA, if feasible. Negative margins: If extensive perineural, large, or named nerve involvement, or if other high-risk features consider adjuvant radiation therapy.

MMS=Mohs micrographic surgery; MCS= micrographically controlled surgery; CCPDMA=complete circumferential peripheral and deep margin assessment.

**Table 3:** Guidelines Recommendations Concerning Lymph Node Surgery of Cutaneous Squamous Cell Carcinoma (cSCC) Patients

**EADO Guidelines [3,6]**	SLNB is currently not recommended in the management of cSCC outside of the setting of clinical trials. A regional therapeutic lymph node dissection should be performed in clinically or radiologically detected lymph node metastasis that is confirmed with cytology or biopsy. The extent of surgical resection is determined by the surgeon in collaboration with the interdisciplinary tumour board.
**NCCN Guidelines [4]**	Discuss and consider SLNB for patients with very-high-risk cSCCs that are recurrent or have multiple risk factors placing them in very-high-risk group and have normal exam of draining nodal basin. Palpable regional lymph node(s) or abnormal lymph nodes identified by imaging studies: Lymph node dissection in operable disease.

SLNB=sentinel lymph node biopsy.
